# Differences in fall injury hospitalization and related survival rates among older adults across age, sex, and areas of residence in Canada

**DOI:** 10.1186/s40621-015-0056-1

**Published:** 2015-09-28

**Authors:** Shanthi Johnson, Sheila Kelly, Drona Rasali

**Affiliations:** 1 Faculty of Kinesiology and Health Studies, University of Regina, 3737 Wascana Parkway, Regina, Saskatchewan S4S 0A2 Canada; 2 Saskatchewan Population Health and Evaluation Research Unit (SPHERU), University of Regina, Regina, Saskatchewan Canada; 3 Population and Public Health, Provincial Health Services Authority, Vancouver, British Columbia Canada

**Keywords:** Falls, Injury hospitalization, Survival rates, Saskatchewan

## Abstract

**Background:**

Falls are the leading cause of injury-related hospital admissions in Canadian older adults, accounting for 85 % of injury hospitalizations among older adults aged over 65 years. While many of these injuries can lead to death, the survival rates of fall-related injuries are rarely examined. This surveillance study examined the fall injury hospitalization and survival rates among older adults in the context of place.

**Methods:**

Saskatchewan’s health administrative data on injury hospitalizations among individuals aged 65 years and over (*n* = 39,867) was utilized for this study. Variables of interest included age group, sex, and the geographical area of residence at the time of hospitalization (rural, urban, north). Logistic regression analysis was applied to determine the association of variables of interest (age group, sex, and area of residence at the time of hospitalization as the covariate) with frequency of fall injury hospitalizations. Probable time to death due to fall-related injury hospitalization was determined by survival analysis.

**Results:**

Three key findings that emerged from the present study are the following: (1) fall injury hospitalizations accounted for 77 % of all injury hospitalizations; (2) fall injury hospitalization rates varied by age group, sex, and area of residence, with advancing age, women, and certain geographical areas showing higher rates; and (3) survival rates also varied by sex and area of residence. Women had longer survival estimates after a fall injury hospitalization compared to men, and those living in the north have the shortest survival estimates.

**Conclusions:**

The findings from the study highlighted the high rate of fall-related injury hospitalization among older adults varying with their age group, sex, and area of residence. These factors need to be considered in injury surveillance and fall prevention research as well as programs and policies that support the reduction of falls.

## Background

Falls are a major threat to the health of older adults in Canada and around the world (Public Health Agency of Canada 2014; World Health Organization 2007). A fall is defined as an individual “unintentionally coming to rest on the ground, floor, or other lower level” (Wolf et al. 1996), which can be with or without injury. In Canada, one in three older adults (over 65 years of age) suffers a fall each year and this figure increases to one in two (50 %) of older adults and those over 80 years of age (Statistics Canada 2014). Falls, which are both predictable and preventable to a large extent, can result in considerable physical, psychological, and economic costs. Approximately 5–25 % of falls result in injuries serious enough to limit normal activities and require hospitalization, such as fractures (Public Health Agency of Canada 2005). Around 85 % of injury-related hospitalizations are caused by fall and about one third of these seniors end up in long-term care (Public Health Agency of Canada 2014) A recent economic burden in direct and indirect costs of injury in Canada report (2015) showed that falls were the leading cause of overall injury cost, accounting for $8.7 billion in 2010 (Parachute 2015). Furthermore, recent reports indicate that falls are the leading cause of injury admissions in Canada’s acute care hospitals, accounting for 85 % of injury hospitalizations among older adults aged over 65 years, 54.4 % of all injury hospitalizations, and 75.7 % of all in-patient hospital deaths (Canadian Institute for Health Information 2010, Statistics Canada 2014). The age-standardized fall-related hospitalization rate was 15.5 per 1000 older Canadian adults in 2008/09 (Scott et al. 2010). As the population ages, this problem is expected to grow and pose an even greater challenge to health services.

Given the public health significance of this issue, epidemiological studies have focused on the rates of falls and injuries among age and sex groups. Research to date suggests a linearly increasing trend in fall and related injuries with age, especially older women having higher rates compared to their male counterparts (Public Health Agency of Canada 2014, Statistics Canada 2014, Hartholt et al. 2011). In addition, crude fall-hospitalization rates have been shown to be higher for women (29.5 per 1000) than men (20.02 per 1000) and also higher with advancing age. Older women in the 65–69 year age group had a fall-hospitalization rate of 5.7 per 1000 compared to 68.6 per 1000 in the 90+ year age group. Similarly, a trend was observed among men with lower hospitalization rates in both age cohorts (4.2 per 1000 in the 65–69 year age group vs. 49.2 per 1000 in the 90+ year age group) (Public Health Agency of Canada 2014). While fall-related injuries can lead to death, the survival rates of fall injuries are seldom examined for age or sex groups. Survival analysis estimates the conditional probability of survival over time from the date of fall injury hospitalization.

While the ability to live in one’s own home as long as possible or *aging in place* is discussed frequently among researchers and policy makers (Lawler 2001), very little research has focused on the context of place or geography as it relates to fall injuries. It is well known that a significantly higher proportion of older adults live in rural areas compared to the overall population. For example, in Saskatchewan, 38 % of the overall population and 58 % of older adults live in rural and remote areas (Statistics Canada 2007). While rural and remote areas such as the northern communities in Saskatchewan are often combined together for statistical purposes, rural and northern communities are considerably different in aspects such as physiographic characteristics and culture. Rural and northern communities may share common attributes such as low population density, distance in time and space for access to health services locally, and the challenges related to the economies of scale in small communities. Specifically, many older adults living in rural and remote communities are faced with a double burden of living with compromised health status at the individual level as well as social isolation and distance in time and space for access to health care and social services at the community or systems level, compared to their urban counterparts (DesMeules and Pong 2006). In the fall prevention context, although falls have been recognized as a significant health issue and considerable advancements have been made in this area of research, regional differences (rural, urban, and northern) in the incidence of fall risks, rates and injuries have not been studied in Saskatchewan. To date, we found only five studies that have conducted urban-rural analysis of fall injuries, in China (Chen et al. 2005), Ireland (Boland et al. 2005), Poland (Wojszel and Bień 2004, Bialoszewski et al. 2008), and Tanzania (Moshiro et al. 2005). These studies reported higher fall rates in rural areas compared to urban areas among community-dwelling independent older adults. In Nova Scotia (Canada), it was found that the fall rates were lower in rural areas compared to urban areas. However, the fall-related hospitalization rates were higher in rural compared to urban areas (Graham 2007). The recent report by Scott et al. (2010) showed that the northern areas (as identified by the territories in Canada) had higher rates (18.3 per 1000) of fall-related hospitalization compared to the national average (15.5 per 1000). None of the studies have compared the rural, urban, and northern areas of residence in fall-related hospitalization rates or the survival rates of those who suffer fall injuries. The high rates of falls among older adults and the vulnerabilities in the rural areas make it critical to provide surveillance data and evidence from which to base innovative solutions to create positive change. In this context, the present study examined the regional differences as defined by rural, northern, and urban areas, as well as the sex and age group differences in fall injury hospitalization and survival rates following hospitalization among older adults in Saskatchewan. This surveillance study was conducted on Saskatchewan residents aged 65 or older who lived in Saskatchewan between 1995/96 and 2004/05. Health administrative database was utilized to analyze relation between frequency of falls and variables of interest (age group, sex, and area of residence at the time of hospitalization as the covariate). We also analyzed the probable time to death due to fall-related injury hospitalization and looked for variations with sex, age, and geographic location.

## Methods

### Study population

Older adults aged 65 and over, determined as of March 31^st^ each year, from the Saskatchewan’s person registry system (PRS), were constituents of the “covered population” in Saskatchewan. The study population includes those with provincial health insurance coverage, living in Saskatchewan anytime during the period from the fiscal year 1995/96 to 2004/05, and were hospitalized with an injury in any year. A fiscal year covers the period from April 1st to March 31st. A detailed exploration of hospital separation data for all older adults hospitalized with an injury (*n* = 39,867) and those with a fall injury hospitalization (*n* = 30,757) was conducted. Information was not available for members of the Royal Canadian Mounted Police (RCMP), members of the Armed Forces and inmates of the Federal Penitentiary and non-Saskatchewan residents, as they were not eligible for provincial health insurance coverage.

### Research design and procedures

This surveillance study used Saskatchewan’s administrative health services data on injury hospitalizations among individuals aged 65 years and over living in Saskatchewan, Canada at any time in the 10-year period from 1995/96 to 2004/05. The Saskatchewan Ministry of Health extracted the injury hospitalization dataset and de-identified before releasing it for this study. De-identified records of injury hospitalizations linked with person registry information on sex, dates of birth, and death available from the Saskatchewan Ministry of Health’s hospital separations data were utilized. To capture all records of injury hospitalizations over the period, the specific diagnostic codes (*ICD-9* and *ICD-10-CA*) based on *International Statistical Classification of Diseases and Related Health Problems* (World Health Organization 2004) were used. Specifically, external causes of injury codes defined the types of injuries recorded in the hospital separation database (Saskatchewan Ministry of Health 2008). All of the ICD codes for external cause of injury (ICD-9 codes 880-889 and ICD-10 CA codes W00 to W19), including those for unintentional falls (E880-E888 and W00-W19), were used. The ICD codes for injuries due to *medical misadventure*, i.e*.,* injuries due to *adverse effects of medical procedure* (E870-E876, E878-E79 and Y60-Y69, Y71-Y84, Y88.1) or *therapeutic agents* (E930-E949 or Y40-Y59 or Y70-Y88) were excluded. Information obtained from the hospital separation dataset included admission and discharge dates, injury hospitalization type (in-patient or day surgery), sex, date of birth, death flag in a given year, date of death, hospital visit type (in-patient or day surgery), dead or alive on discharge, length of stay, age, ICD-codes for falls, and most responsible diagnosis for the hospital stay.

### Variables of interest

The variables of interest for this analysis were type of injury, cause of injury, age group, sex, area of residence within the province (rural, urban, and north), and year of injury hospitalization. Specifically, age groups (65–74, 75–84, and 85+ years), sex (male and female), and the area of residence at the time of injury (rural, urban, north) were selected as categorical variables for analysis. Urban area of residence included all of Saskatchewan’s 13 cities (Estevan, Humboldt, Melfort, Melville, Moose Jaw, Lloydminister, North Battlefords, Prince Albert, Regina, Saskatoon, Swift Current, Weyburn, and Yorkton) with a total population of 78,642 older adults (65+) in 2001, the year for which the covered population was considered as a proxy for average population for use as the denominator for average of all 10-year data. The northern area of residence encompasses the northern health regions of the province (Keewatin Yatthé, and Mamawetan Churchill River Regional Health Authorities, and Athabasca Health Authority), each with their own unique demography and health issues (Saskatchewan Ministry of Health 2008). For the purpose of this study, except for northern region identified as northern area, all cities in Saskatchewan represented the urban area, while rural area of residence included the rest of Saskatchewan comprising towns, villages, and farms.

### Data analysis

Records of all individuals aged 65 and over hospitalized with an injury (*n* = 39,867) were extracted from the dataset and classified into a category of “fall injury” (1 = yes, 0 = no). Descriptive analysis of fall injury hospitalizations (*n* = 30,757) by age group, sex, and area of residence was conducted. Crude fall injury hospitalization rates were calculated as the number of injuries by fiscal year (both sexes) divided by the corresponding covered population (aged 65 and over) in Saskatchewan. The covered population for the year 2001 was selected as the common denominator because it was roughly in the middle of the period of review in the present study.

Logistic regression analysis was applied to all injury hospitalizations (*n* = 39,867) with a fall (yes = 1) or without a fall (no = 0) in order to determine if the frequency of fall injury hospitalizations was associated with the following variables (risk factors): age group, sex, and area of residence at the time of hospitalization as the covariate. A stepwise logistic regression analysis was conducted on all fall-related injury hospitalizations. Stepwise logistic regression analysis was selected as it conducts forward selection or backward elimination of the factors as required for the regression model used. Likelihood ratios were used to determine the overall model significance of final regression models. Odds ratios (OR) and 95 % confidence intervals (CI) were presented for significant risk factors. Somer’s D goodness of fit test was conducted to assess the strength of pairs within the model. Strong associations among the variables were highlighted for the regression analysis. A *p* value ≤0.05 was considered significant for all statistical analysis (Lang and Secic 2006).

A survival analysis of person-level data by year was conducted, using SAS LifeTest Procedure that produces life tables and Kaplan-Meier survival curves, to determine the probable time to death (event) for those who experienced a fall injury hospitalization for the first time. By selecting the first record of injury hospitalization for a patient in each year, the duplicate records are removed for the calculating the person-level fall injury hospitalization rates and conducting survival analysis. Survival analysis estimates the conditional probability of survival over time from the discharge date of fall injury hospitalization in the database. The analysis accommodates right-censored data where the end-point event has not yet occurred or is unknown (Laurent 2002). The percentage of older adults still alive at the end of each time interval (every 5 years after age 65) was used to estimate the probability that a typical older adult will be alive at the end of any given period. When graphed, these estimates formed distribution (Kaplan-Meier) curves of the probabilities of survival for the different time periods. Life tables present the number of failed, censored, effective sample size and hazard ratios (probability per time unit that a case that has survived to the beginning of the respective interval will fail in that interval) for older adults hospitalized with a fall injury (Lang and Secic 2006). Only the first instance of fall injury hospitalization ever was included in each analysis to avoid duplication of those who might have had more than one fall injury hospitalization over a 10-year study period. The case definition for the survival analysis was the first hospitalization for a fall injury over 10 years under review (analysis reflects impact of only first fall injury; not the possible multiple fall injuries in database for a given individual). Kaplan-Meier survival analysis curves were presented by age, sex, and area of residence and overall (mean) survival for each analysis. Log-rank and Wilcoxon tests of homogeneity were conducted to assess differences among strata for each risk factor (Lang and Secic 2006). All analyses were performed using SAS software, Version 8 (SAS Institute, Inc. Cary, NC, USA).

## Results

Data retrieval yielded 39,867 injury hospitalizations among Saskatchewan older adults over the 10-year period from 1995/96 to 2004/05. Of all the injury hospitalizations extracted, unintentional falls accounted for the majority at 77 % (*n* = 30,757) injury hospitalization.

Table 1 provides descriptive frequencies for those older adults with a fall injury hospitalization. The covered population data for Saskatchewan adults over age 65 (*n* = 148,032) for the year 2001 that represents the mid-point of the time frame in this analysis are presented. The number of patients with fall injury hospitalization is given by risk factor: age group, sex, and area of residence. Those in the 75–84 year age group had the highest percentage (40.4 %) of the fall injury hospitalizations followed by the 85 years and up age group and the 65–74 year age group. As expected, fall injury hospitalizations among women were more than double the numbers of men (69.9 % vs. 30.1 %). Older adults residing in rural areas experienced 52.8 % of the fall injury hospitalizations, which ranked higher than those in urban areas (46.5 %) and north areas (0.7 %) of Saskatchewan, respectively. Crude numbers of fall injury hospitalizations were consistent with slight fluctuations across years from 3029 (9.8 %) to 3244 (10.6 %) over the period.

**Table 1:** A 10-year distribution and annual average crude rate of fall injury hospitalizations in Saskatchewan seniors, by age group, sex, and area of residence, 1995/96–2004/05

Characteristics	Covered population in 2001 *N* = 148,032	Population with fall injury hospitalizations in 10 years *n* = 30,757	Annual average crude rate of fall injury hospitalization per 1000
Age group			
65–74 years	72,686	6503 (21.1)	89.5
75–84 years	53,844	12,420 (40.4)	230.7
85+ years	21,462	11,834 (38.5)	551.4
Sex			
Female	83,560	21,512 (70.0)	257.4
Male	64,472	9245 (30.0)	143.3
Area of residence			
Rural	64,075	16,247 (52.8)	253.6
Urban	78,642	14,296 (46.5)	181.8
North	5315	214 (0.7)	40.3

Percentages in the parentheses are based on total *n* within each group

The logistic regression included all of the injury hospitalizations for falls (*n* = 30,757). Among the 30,757 hospitalizations of older adults with at least one fall injury hospitalization over the 10-year period, a model was found to attribute the occurrence of fall injury hospitalizations to all three risk factors included in the final model.

Table 2 provides results from the logistic regression analysis for the risk factors associated with fall injury hospitalization. Factors that were found statistically significant in attributing to a fall injury hospitalization at *p* < 0.0001 were age group, sex, and area of residence. The odds of having a fall injury hospitalization were lowest among those in the 65–74 year age group (OR: 0.26; 95 % CI: 0.24–0.28) and increased with advancing age up to the reference category 85+ year age group. The odds of having a fall injury hospitalization were higher in women (OR: 2.15; 95 % CI: 2.05–2.26) than in men. The odds of a fall injury hospitalization were lowest among those in the north area of residence (OR: 0.58; 95 % CI: 0.46–0.73) and were higher in rural and urban areas, respectively. The rural-urban differences in the odds of a fall injury hospitalization were significant (*p* < 0.05) with odds ratio being lower in rural areas compared to the reference category urban areas. Fiscal year variation served as a controlling variable (covariate) in the model. The Wald test of the global null hypothesis was 2949.26, *p* < 0.0001. For the fall injury hospitalization, global models were all statistically significant and the goodness of fit tests indicated a consistently good fit model. Somer’s D, a test of the strength and direction of relationships between pairs (range from −1.0) where all pairs disagree to 1.0, where all pairs agree, was 0.4 for the model 19. From Table 2, it was evident that the odds of fall injury hospitalization were highest for women, those in the oldest age group (85+ years), and those in urban areas, compared to their respective counterparts.

**Table 2:** Stepwise logistic regression model for risk factors associated with fall injury hospitalizations among older adults in Saskatchewan, 1995/96–2004/05 (*N* = 30,757)

Risk factor	SE	Wald *χ* ^2^	Odds ratio (OR)	95 % CI	*p* value
Age group					
65–74 vs. 85+ years	0.0178	1331.78	0.26	0.24–0.28	<0.0001
75–84 vs. 85+ years	0.0170	7.57	0.48	0.45–0.51	0.0059
Sex					
Female vs. male	0.0126	928.17	2.15	2.05–2.26	<0.0001
Area of residence					
North vs. urban	0.0774	16.71	0.58	0.46–0.73	<0.0001
Rural vs. urban	0.0405	4.87	0.87	0.83–0.92	0.0273

*SE* standard error, *OR* odds ratio, *CI* confidence interval

Further to examining the odds of fall injury hospitalization based on age group, sex, and area of residence, survival analysis was performed to determine the probability of the time to death (mortality) related to first fall injury hospitalization in all persons (*n* = 30,456). Only the first instance of fall injury ever over the 10-year period was included to reflect its effect on the probability of survival after a fall injury hospitalization. A statistical model was created to estimate the risk of death associated with risk factors for fall injury hospitalization adjusted for age (5-year intervals). The risk factors examined were sex and area of residence (Figs. 2 and 3) against the age curve. Kaplan-Meier survival curves are presented by overall (mean) survival and by risk factor for fall injury hospitalization. Results describe the survival experience for the older adults reviewed over the period. The 95 % confidence level hazard rates for fall injuries (*N* = 30,456) increased from 0.002 to 0.200 over time. Hazard rates indicate the probability that a case which has survived to the beginning of a respective time interval will fail to survive in that interval (Lang and Secic 2006). Survival differences for each stratum within each risk factor for fall injury hospitalization were compared. Tests of equality over the strata for each risk factor showed they were significant at *p* < 0.0001 with a 95 % CI for those with a fall injury hospitalization.

**Fig. 1:**
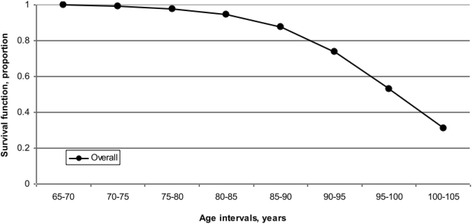
Overall survival curve for older adults (*n* = 30,456) with first fall injury hospitalizations over a 10-year period, Saskatchewan, 1995/96–2004/05

**Fig. 2:**
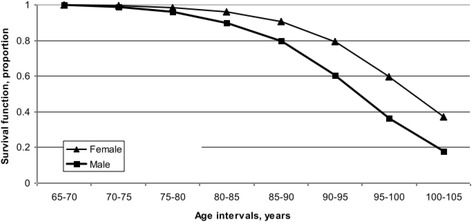
Survival analysis for older adults (*n* = 30,456) with first fall injury hospitalization over a 10-year period, by sex, Saskatchewan, 1995/96–2004/05

**Fig. 3:**
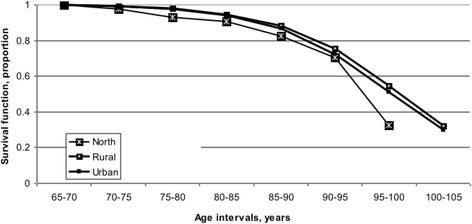
Survival analysis for older adults (*n* = 30,456) with first fall injury hospitalization over a 10-year period, by area of residence, Saskatchewan, 1995/96–2004/05

Figure 1 estimates the overall (mean) survival time for those older adults with a fall injury hospitalization. The overall survival time after the first fall injury hospitalization reflects the mean time to death following the injury hospitalization. Figure 2 shows the survival time by sex; women had longer survival estimates after the first fall injury hospitalization, compared to men. Between the ages of 65 and 80, both men and women had similar survival estimates. Beyond 80 years of age, women had higher survival estimates than men, with this tendency continuing beyond 105 years where the survival curve converged to each sex. Sex differences played a role in the estimated length of survival after a fall injury hospitalization. Figure 3 shows the survival time by area of residence; those living in rural and urban areas of Saskatchewan at the time of a fall injury hospitalization had the longest survival estimates (up to 110 years) while those residing in north areas had the shortest (up to 105 years). The survival rates were similar for residents in rural and urban areas between 65 and 85 years. Beyond 85 years, urban residents had lower survival estimates compared to their rural counterparts. Area of residence affected overall survival estimates for older adults in Saskatchewan. Results of the log-rank and Wilcoxon tests for homogeneity for each risk factor for those with a fall injury hospitalization are found in Table 3. The log-rank and Wilcoxon tests indicated a significant difference over time with *p* < 0.001 between strata.

**Table 3:** Survival tests for homogeneity in fall injury hospitalizations among older adults in Saskatchewan, 1995/96–2004/05 (*N* = 30,456)

Factor	Log-rank *χ* ^2^	Wilcoxon *χ* ^2^	*p*-value
Sex	753.76	675.68	<.0001
Area of residence	24.45	18.75	<.0001

## Discussion

The foremost consideration from the present study is that three fourths of all injury hospitalizations among older adults are the result of falls. The literature reports 5–25 % of falls result in serious injuries such as fractures requiring hospitalization. In addition to the high fall hospitalization rates reported in this study and elsewhere (Public Health Agency of Canada 2005), Scott et al. (2010) have shown that the length of stay for fall injury is 70 % more than all other hospitalizations. Not surprisingly, the economic burden of falls has been reported to be $8.7 billion Canadian dollars in 2010 in Canada and $319 million in Saskatchewan (Parachute 2015). Given that falls are common and costly, preventing fall injuries is an effective way to take some of the pressure off of the health care system and reduce the pain and suffering associated with these injuries.

The present study is the only study known to us to date to examine injury hospitalization and survival rates by areas of residence of rural, urban, and northern Saskatchewan. This surveillance study used the Saskatchewan’s health administrative data on injury hospitalization and mortality rates among older adults aged 65 years and over living in the province of Saskatchewan, Canada over a 10-year period. The present study provides insights and highlights the need for falls and injury surveillance. Three key findings emerged from the present study: (1) In the 10-year time period, 30,757 fall injury hospitalizations were recorded, accounting for 77 % of all injury hospitalizations in older adults aged 65 or older. This indicates the need to prevent falls among older adults as a way to reduce the burden of falls on the health care system. (2) In addition to substantiating the age and sex differences in fall injury hospitalization rates reported in the literature (Scott et al. 2010), the present study showed geographical differences. Although the fall injury hospitalization rates were the lowest in the north, the data should be interpreted with caution given the small numbers. The difference in fall injury hospitalization rates raises the issue of place or area of residence being a risk factor. (3) The survival analysis, which provides the probability estimates of time to death following injury hospitalization, showed that women had longer survival estimates after a fall injury hospitalization compared to men. Furthermore, area of residence affected overall survival estimates for older adults in Saskatchewan, with those living in the north having the shortest survival estimates. Rural and urban residents had similar survival estimates until age 85, with urban residents having lower survival rates beyond this age.

Consistent with the literature, the present study showed that the odds of having a fall injury hospitalization were higher for older women and individuals with advancing age. The role of area of residence in terms of rural, urban, and northern areas is seldom examined. As indicated earlier, studies have found higher fall injury rates in rural areas compared to urban areas (Chen et al. 2005, Boland et al. 2005, Wojszel and Bień 2004, Bialoszewski et al. 2008, Moshiro et al. 2005). Although Graham (2007) reported higher fall injury rates in rural areas than their urban counterparts in Nova Scotia, the rates were reversed for fall injury hospitalization with urban areas reporting higher rates. In the present study, the odds ratio for fall hospitalization from the logistic regression analysis was higher in urban areas with the reverse observed in the descriptive average crude rates reported for per 1000 population. The differences between fall rates and fall hospitalization rates in rural and urban areas suggest the need for further research into the nature of injuries and other influencing factors as well as the statistical techniques used. In a recent study, Scott et al. (2010) showed that the northern areas (as identified by territories in Canada) had higher rates (18.3 per 1000) of fall-related hospitalization compared to the national average (15.5 per 1000). Yet, in the present study, the rates of fall injury hospitalization were the lowest in northern areas. Further research is needed to examine the underlying reasons for regional or geographical differences, whether they are related to the nature of injury itself (e.g., severity) or systemic issues (e.g., limited access to primary care contributing to higher level of care). It has been well documented that rural communities are faced with challenges associated with the availability and access to health and social services (Laurent 2002). Asthana and Halliday (2004) suggest that “because rural areas have less chance of achieving economies of scale than their urban counterparts, they must either develop more numerous smaller units (which has significant cost implications) or sacrifice accessibility by developing large distances between service users and service centers.” The latter option routinely transfers costs to patients and care givers with consequent equity issues, particularly for ‘transport poor’ groups such as older adults especially those in low socioeconomic status who have limited mechanical transportation options available to them. The difference in fall injury hospitalization rates raises the issue of place or area of residence as a risk factor. When promoting innovations in community health, the National Rural Health Strategy (Society of Rural Physicians of Canada 2010) calls for applying a “rural lens” or the view point and experiences of rural residents to examine significant health issues. Rural and remote areas, by virtue of necessity, have to explore efficient and effective mechanisms to provide care and support collaborative models of health.

Another key finding emerging from this study relates to the survival rates of older adults after fall injury hospitalization. This project is unique in that it included survival analysis rarely conducted in injury hospitalization studies. Identification of risk factors affecting overall survival after a fall injury hospitalization should lend itself to enhancements in screening and preventive techniques (Public Health Agency of Canada 2014, Aharonoff et al. 1998, Michaelsson et al. 2005). In the present study, the longest survival estimates were observed for women, compared to men. Although men are known to have lower rates of falls and lower fall injury hospitalization rates (Public Health Agency of Canada 2005), their lower survival rates following fall injury hospitalization could be a factor contributing to the overall lower life expectancy of males compared to females (Statistics Canada 2013). Area of residence seemed to influence the probability of survival resulting from fall injury, although this should be interpreted with caution due to small numbers in the north, and this issue should be examined further. The lower survival rates among those with fall injury hospitalization in the north may account for, at least partly, the lower life expectancy reported in the north as compared to other health regions (Statistics Canada 2013) and could be related to lack of or limited access to timely and adequate health care (Hay et al. 2006). However, the link between access to health care for fall injuries and survival rates must be examined in the future. Studies have shown that health status decreases as one travels to more remote regions and with vulnerable groups such as the older adults most affected (DesMeules and Pong 2006). It is also well known that poor health indicators and status contribute to the vulnerability of older adults to adverse health outcomes such as falls (DesMeules and Pong 2006, Mitura and Bollman 2003). While rural and northern areas are often combined for statistical purposes, the findings suggest the need to examine these two areas as distinct groups. In addition, the difference in fall injury hospitalization and survival rates raises the issue of place or area of residence as a risk factor.

The present study has several strengths. This is the first study to utilize health administrative data over a 10-year period to examine survival rates related to the fall injury hospitalization rates. As such, it has provided a more accurate representation of injury in the province than self-reported data from previous injury studies. In addition, internationally recognized ICD codes for classification were used in this study (World Health Organization 2004). Finally, this project provides population-based evidence generated using the administrative data from the Saskatchewan Ministry of Health. The findings from the study also highlight the need for further research as well as opportunities to prevent falls as a method for reducing hospitalization rates in older adults.

While the study has shed some light on the differences in fall injury hospitalization and survival rates, the limitations inherent in the research design and data used should be acknowledged. The present study used health administrative hospital data from over a 10-year period. Lack of control over initial database entries in the Saskatchewan Ministry of Health’s hospital separations database may have been an issue with the possibility of coding errors (e.g., spontaneous fractures as a result of advanced osteoporosis resulting in falls). Also, hospitalization data were not available for the following individuals residing in the province: members of the RCMP, members of the Armed Forces, and inmates of the Federal Penitentiary. However, these accounts for less than 1 % of the population and this should have had little effect on this study. In addition, the total number of fall hospitalizations is generally influenced by the size of the population within a geographical jurisdiction. In Saskatchewan, the urban area of residence had the highest population, followed by rural and northern areas. As such, the fall hospitalization crude rates per 1000 population are reported for the group comparison. The change from ICD-9 to ICD-10-CA codes in the year 2000 through 2002 may have affected the coding of data in hospitals and the data on fall injuries. In addition, while the survival analysis showed the probability of death resulting from a fall injury hospitalization within the study context (within Saskatchewan and among older adults), the data analysis does not allow comparison of the findings with the general population of older adults in Saskatchewan without a fall injury hospitalization. This study also did not control for any lifestyle or environmental factors and the differences in these factors among the age, sex, and geographical location groups. For example, regional difference may be a function of survival rates in their population and the physiological capacity to meet minimal functional threshold for survival.

The study creates several propositions for future studies. It is important to examine whether the nature of fall injury and its level of seriousness contributed to the lower survival rates among men. It may be possible that, although men fall less frequently than women, the nature of the injuries sustained may be more serious. It also raises questions about the health-seeking behaviors among men and women. It may be possible that men do not seek care until more serious injuries occur (Galdas et al. 2005). Furthermore, the impact of area of residence is a rarely researched area that should be given more attention in future studies, as it seems to influence the probability of survival following a fall injury. The residents of northern Saskatchewan showed lower survival rates than their rural and urban counterparts despite displaying lower rates of fall-related injury hospitalizations. Future studies in this area should explore whether this low rate of survival in the north may contribute to the lower life expectancy or whether it could be related to lack of or limited access to timely and adequate health care.

## Conclusions

A study on Saskatchewan residents aged 65 or older showed a high rate of fall-related injury hospitalization, where 77 % of the older adults experienced a hospitalization due to fall-related injury over a 10-year period. The injury hospitalization rate was higher in people older than 75 years and the survival rate after injury hospitalization decreased with increasing age. The rate and risk of fall injury hospitalization were higher in women, however, post-injury survival estimates were higher in women than man. The rate and risk of hospitalization were highest among the rural residents, nevertheless, the survival estimates were similar for rural and urban residents.

## Abbreviations

CIHI: Canadian Institute for Health Information
CI: confidence intervals
ICD: international classification of diseases
OR: odds ratios
RCMP: Royal Canadian Mounted Police
PRS: Saskatchewan’s person registry system
SPHERU: Saskatchewan Population Health and Evaluation Research Unit
